# Understanding the substance use of autistic adolescents and adults: a mixed-methods approach

**DOI:** 10.1016/S2215-0366(21)00160-7

**Published:** 2021-08

**Authors:** Elizabeth Weir, Carrie Allison, Simon Baron-Cohen

**Affiliations:** a Autism Research Centre, Department of Psychiatry, University of Cambridge, Cambridge, UK

## Abstract

**Background:**

Autistic individuals might be more likely to misuse substances than non-autistic individuals. Better understanding of these patterns can help clinicians identify strategies to reduce substance use, protecting physical and mental health. The aim of this study was to compare the experiences of substance use between autistic and non-autistic adolescents and adults.

**Methods:**

This study is a mixed-methods study, including both quantitative (closed-ended questions) and qualitative (one open-ended question) online assessments. Data were collected as part of a larger study, the Autism and Physical Health Survey, in which we administered an anonymised, online questionnaire to autistic and non-autistic individuals aged 16–90 years. In the present study, we investigated data on substance use or misuse, using two overlapping but separate samples from the survey (one sample with complete quantitative responses and one sample with complete qualitative responses). Binary measures of substance use were investigated using unadjusted and adjusted binomial logistic regression models. Content analysis was used to compare experiences of autistic and non-autistic adolescents and adults. We used Fisher's exact tests to assess differences in frequency of reporting particular qualitative themes and subthemes.

**Findings:**

Survey recruitment was done between Feb 7, 2018, and Aug 26, 2019. At the end of the recruitment, 3657 individuals had accessed the survey. After excluding duplicates as well as participants with missing or incomplete responses, we had data from 2386 participants (1183 autistic and 1203 non-autistic participants; 1571 female and 815 male participants) for the quantitative analyses and data from 919 participants (429 autistic and 490 non-autistic participants; 569 female and 350 male participants) in the qualitative analyses. The samples for the quantitative and qualitative analyses were predominantly composed of female individuals, White individuals, UK residents, and those without intellectual disability. Autistic individuals were less likely than non-autistic individuals to report consuming alcohol regularly (16·0% of autistic individuals *vs* 22·2% of non-autistic individuals; adjusted model: odds ratio [OR] 0·69, 95% CI 0·55–0·86; p=0·0022) or binge-drinking (3·8% *vs* 8·2%; adjusted model: OR 0·38, 0·26–0·56; p<0·0001). Autistic male participants were less likely than non-autistic male participants to report ever having smoked (50·8% of autistic male participants *vs* 64·6% of non-autistic male participants; adjusted OR 0·50; 0·32–0·76; p=0·0022) or ever using drugs (35·4% *vs* 52·7%; adjusted OR 0·53; 0·35–0·80; p=0·0022). Regarding our qualitative analyses, among participants who reported a specific motivation for drug use, compared with non-autistic individuals, autistic individuals were nearly nine times more likely to report using recreational substances to manage behaviour (OR 8·89, 2·05–81·12; p=0·0017) and more likely to report using recreational substances to manage mental health symptoms (OR 3·08, 1·18–9·08; p=0·032). Autistic individuals were also more likely to report vulnerability associated with substance use (OR 4·16, 1·90–10·05; p=0·00027), including childhood use of drugs and being forced or tricked into using drugs.

**Interpretation:**

Autistic individuals might be less likely than non-autistic individuals to report engaging in substance misuse. They also report using drugs to self-medicate. Clinicians should be aware of vulnerability linked to substance use among autistic patients and should work cooperatively with patients to effectively manage autistic and comorbid symptoms.

**Funding:**

Autism Research Trust, Rosetrees Trust, Cambridge and Peterborough NHS Foundation Trust.

## Introduction

Autism spectrum conditions (henceforth autism) are a group of lifelong, neurodevelopmental conditions denoted by social and communication difficulties, repetitive behaviours, restricted interests, and variances in cognitive profile, including atypical sensory experience and information processing, motor abilities, and intellectual ability.^1^ 1–2% of the general population are diagnosed as autistic^2^ and male individuals are diagnosed three to four times more frequently than female individuals.^2^, ^3^ There is a growing body of evidence to indicate that autistic female individuals might be underdiagnosed owing to a different presentation of autism symptoms, higher rates of internalising difficulties, and higher rates of camouflaging.^4^, ^5^, ^6^, ^7^ Camouflaging can be defined as altering one's behaviour or personality traits to align with social norms; and compensation (a related but distinct concept) can be understood as conscious or subconscious processes that allow individuals with neurodevelopmental conditions (eg, autism) to reduce the presentation of their symptoms to others, despite ongoing difficulties. Autistic individuals might attempt to use strategies of camouflaging or compensation to minimise or obscure their autism symptoms to align with societal expectations of behaviour;^5^, ^8^ however, this behaviour can come at huge cost, worsening mental health problems and even increasing risk of suicidality.^4^, ^5^, ^8^, ^9^ Please note that we use identity-first language (eg, autistic individual) throughout the manuscript, as this terminology is preferred by the majority of the autistic community in the UK.^10^

**Research in context**

**Evidence before this study**

We searched PubMed and Google Scholar using various combinations of the search terms “autis*”, “substance use”, “substance misuse”, “dependence”, “addiction”, “quantitative”, “qualitative”, and “adult” with no language restrictions for all studies from database inception until Nov 1, 2017, before beginning the study, and again from database inception until Feb 11, 2021, after study completion. Existing studies vary greatly in size and scope. Multiple large population-based studies suggest that autistic individuals have an increased risk of substance misuse or misuse. Several qualitative studies with small sample sizes have been collectively described in reviews and meta-analyses. They have identified motivations, protective factors, and risk factors for substance use or misuse among autistic individuals; however, none of these studies have attempted to quantify the relative likelihood of autistic and non-autistic adolescents and adults reporting these behaviours or experiences.

**Added value of this study**

This mixed-methods study provides new evidence of differences in smoking and alcohol use, as well as differences in motivations for substance use, among autistic and non-autistic adolescents and adults. This analysis includes large samples of autistic female individuals and older autistic adults, which are groups that remain neglected in research. Our findings show key information about sex differences, highlighting that autistic male individuals were less likely to ever have smoked or engaged in recreational drug use than non-autistic male individuals, whereas there were no significant differences between autistic and non-autistic female groups. Qualitatively, our results suggest that autistic individuals were more likely to report using substances to manage behavioural symptoms (including autism symptoms) and using them to manage mental health symptoms than were non-autistic individuals. The findings also provide evidence for the reduced likelihood of reporting social motivations for drug use among autistic individuals compared with non-autistic individuals. New areas of self-reported vulnerability have been identified by this study, including childhood use of drugs and being forced or tricked into using drugs.

**Implications from all the available evidence**

Health-care providers should work with autistic people to identify and effectively manage the autistic symptoms as well as the comorbid behavioural, mental, and physical health symptoms that require additional support, to prevent self-medication and possible substance misuse. Clinicians should be aware of increased risk of adverse life events for autistic individuals, some of which might be connected to substance use. This study reaffirms the importance of early autism diagnosis and supportive health care across the lifespan.

Several studies taken from both clinical and general population samples (with sample sizes ranging from 89 to 4123 autistic participants) suggest that autistic individuals are less likely to smoke, use tobacco, use nicotine,^11^, ^12^, ^13^ or misuse substances (including alcohol) than non-autistic individuals.^12^, ^13^, ^14^, ^15^, ^16^ By contrast, results from larger studies and one systematic review indicate that autistic individuals might have an increased likelihood of developing substance use-related problems.^17^, ^18^, ^19^, ^20^ Specifically, in large population-based studies in Sweden (including 26 986 autistic individuals) and Norway (including 7528 autistic individuals), autistic individuals were twice as likely to have substance use problems than non-autistic individuals; and even their non-autistic siblings and parents were at increased risk of substance use problems compared with controls, suggesting that genetic or environmental factors might contribute to risks.^17^, ^19^ Additional diagnoses of ADHD and intellectual disability seem to moderate the risk of substance misuse, with an ADHD diagnosis increasing the risk and a diagnosis of intellectual disability decreasing the risk.^17^, ^18^, ^19^ Existing studies indicate that participants with ADHD, ADHD and autism, as well as those with other developmental disorders were all at greater risk of substance use problems than the autistic participants, making it difficult to quantify the risk of substance use or misuse that is specific to autism—even in large, population-based samples.^17^, ^19^, ^20^

Several population-based studies of neurotypical adults suggest that substance use or misuse (including alcohol, tobacco, prescription and recreational drugs) is associated with many physical health risks, including respiratory problems, cancer, heart disease, hypertension, heart attack, stroke, reproductive morbidity, diabetes, liver damage or disease, and sleep conditions.^21^ In a previous study that we did with the same sample as the current study, we found that substance use was not associated with increased health risks among the autistic individuals;^22^ however, this study only used current alcohol consumption and greatest smoking frequency as covariates, which does not capture all aspects of substance use. In addition to physical health risks, substance misuse might negatively affect the quality of life of autistic individuals and exacerbate existing difficulties with functional outcomes, such as maintenance of employment and education.^18^

Six studies have attempted to establish motivations, protective factors, and risk factors for substance use or misuse using qualitative methods;^18^, ^23^ however, these studies included small samples of autistic individuals (n<50) and even fewer autistic females (n≤16). Autistic individuals were more likely to use substances to compensate for comorbid mental health conditions (as well as psychological distress) and perceived social difficulties; weak executive functioning, maladaptive coping style, late autism diagnosis, few social resources, lack of structure in daily life or leisure activities, family history of substance misuse, early smoking onset, and adverse childhood experiences were additional risk factors for substance use or misuse.^18^, ^23^

Only two studies considered sex or gender differences in substance misuse.^14^, ^19^ In the general population, male individuals are far more likely to use and misuse substances than female individuals;^24^ yet, this pattern appears more complex among autistic individuals, with smaller differences between male and female individuals.^14^, ^19^ Crucially, none of these studies consider sex or gender differences in substance use patterns or in qualitative studies when considering motivations, protective factors, or risk factors for substance use or misuse.

Research into the substance use of autistic individuals is limited in sample size and scope; however, it is clear that differences in substance use might leave autistic individuals susceptible to wide-ranging negative consequences regarding daily functioning and physical health. The present study attempts to explore whether there are quantitative or qualitative differences in substance use between autistic and non-autistic adolescents and adults.

## Methods

### Study design and participants

This study is a mixed-methods study, including both quantitative (closed-ended questions) and qualitative (one open-ended question) online assessments. Data were collected as part of a larger study, the Autism and Physical Health Survey (APHS). Any consenting individual of at least 16 years of age was eligible to participate in the APHS. To include as many relevant participant records as possible, the quantitative and qualitative analyses of our study include two different but overlapping samples—one sample with complete quantitative responses and one sample with complete qualitative responses—both taken from the APHS dataset. We used an anonymous, self-report, cross-sectional survey in English via the website Qualtrics to collect data regarding demographics, autistic traits (we used a short version of the Autism Spectrum Quotient, AQ-10, administered to non-autistic participants only), lifestyle-related factors (including both quantitative and qualitative substance use information), personal medical history, and family medical history. We used a convenience sampling framework to recruit participants via the Cambridge Autism Research Database, Autistica's Discover Network, autism support groups and charities, as well as social media (especially Twitter and Facebook). Because of our recruitment strategies, our control sample might have been biased towards individuals with an interest in autism or those with undiagnosed autism. In an attempt to recruit a general population sample, we advertised the study via Facebook and did not target specific autism groups or forums. All advertisements encouraged participation from both autistic and non-autistic adolescents and adults.

All questions related to substance use were developed by our research team by consulting publicly available information and questions from surveys from the UK National Health Service, UK National Institute for Health and Care Excellence, US National Institutes of Health, and WHO. All substance use data were collected as part of APHS, with the dual purposes of describing the substance use or misuse of autistic individuals directly, while also considering the relationships between substance use and risk of physical health conditions among autistic individuals.^22^ The present analyses used binary measures of smoking and alcohol use (the binarisation was done before looking at the results). These analyses also incorporated measures of recreational drug use and second-hand smoke exposure (which were originally measured as binary [yes or no] responses). Analysing the data in this binarised structure allowed us to establish cutoff points for possible substance misuse (eg, ≥5 alcoholic drinks per average session, consumption of alcohol on ≥3 days per week) rather than describing substance use more generally, as the binarised data might have greater clinical relevance. As questions relating to substance use might be considered sensitive to some participants, we made all questions optional, and participants were informed of this at the beginning of this section of the survey. We had high response rates (>98·99%) to all quantitative questions. The specific phrasing of all relevant questions have been provided in the appendix (pp 5–8).

This study obtained ethics approval from the University of Cambridge Human Biology Research Ethics committee (HBREC.2017.28). Written, online informed consent was obtained from all participants. The study protocol has been included in the appendix (pp 12–21).

### Procedures

The qualitative section of the survey relied on responses to a single question related to substance use. All participant records without a response to this question were excluded from the qualitative sample. Then, all suspected duplicate records were eliminated from the quantitative and qualitative samples. As we did not collect any personally identifiable data, we developed an algorithm to exclude potential duplicate responses. Any responses that matched a previous response across the following 11 criteria were excluded: autism diagnosis (yes/no), specific autism diagnosis, type of diagnosing practitioner, year of autism diagnosis, country of residence, sex assigned at birth, current gender identity, education level, age, maternal age at birth, and paternal age at birth. To provide the most conservative analysis, we did not exclude individuals with a high autism quotient score from the control group.

The autistic cohorts of each sample included all individuals who self-reported an autism diagnosis made by a medical practitioner. As the survey was anonymous, participants did not provide diagnostic assessments; yet we required that they disclose additional information to verify their diagnosis: type of practitioner who diagnosed them, the year of their diagnosis, their specific diagnosis, and whether they have a syndromic form of autism. Individuals whose autism status could not be confirmed were excluded from both the autistic and control cohorts of the quantitative sample. By contrast, the qualitative sample included individuals who self-diagnosed as autistic, suspected autism, or were awaiting autism assessment; they were included in either the autistic or non-autistic cohorts on the basis of their own designation for autism diagnosis. Although this choice makes it possible that the qualitative section of our study underestimates true group differences in the frequency of reporting particular codes, subthemes, or themes between autistic and non-autistic individuals, we felt it was important to explore the real-world experiences and opinions of self-diagnosed and undiagnosed individuals.

The non-autistic group of the quantitative sample included individuals who provided relevant responses without an autism diagnosis and for whom an autism diagnosis or duplicate response were not suspected. The non-autistic group of the qualitative sample included all non-duplicate individuals who provided a relevant response and self-identified as non-autistic (even if autism was suspected or the individual was awaiting autism diagnosis).

Sex assigned at birth was self-reported as female, male, or other; current gender identity was also recorded but is not included as part of this analysis. Education level was coded as a categorical variable and used as a proxy measure of socioeconomic status; it was defined as the highest qualification held with the following options: no formal qualifications, secondary school or high school level qualifications, further vocational qualifications, university undergraduate level qualifications (BA, BSc, etc), and university postgraduate level qualifications (MA, MSc, PhD, Certificate, etc). Because of low response rates of individuals from non-White ethnic backgrounds, we used a binary representation of ethnicity (White *vs* non-White) in all of our analyses. Descriptive breakdowns of ethnicity for both the quantitative and qualitative samples are provided in the appendix (p 5). We derived a categorical variable of country of residence based on frequency with the following options: UK, USA, Germany, Australia, and other.

We included one open-ended, free-text question, namely: “Please list any recreational substances/drugs you have used and how long you used them for. Please provide any information that you think may be relevant.” With this question, we intended to give autistic and non-autistic individuals the opportunity to provide information related to their experiences of substance use organically and to explore various topics without explicit direction of themes from the questionnaire. We used the content analysis method, because it flexibly uses some principles of thematic analysis while also providing the opportunity to quantify the relative frequency of reporting of specific code-level and thematic data.^25^, ^26^, ^27^ As the analysis was intended to be exploratory in nature, EW developed codes inductively to answer two research questions: (1) what experiences or themes related to substance use do autistic and non-autistic individuals choose to discuss without explicit direction, and (2) are there differences in the relative frequency of discussion of these themes between autistic and non-autistic individuals. EW organised the codes into an original schema with possible themes and subthemes identified on the basis of the explicit, word-level information from the participants (rather than at the latent, interpretive level); all authors then discussed and clarified these themes or categories and established the final structure together.

### Statistical analysis

We used R version 3.6.2 to do all quantitative analyses. We employed both unadjusted and adjusted models (binomial logistic regression using the “glm” function from the “stats” package); the adjusted model controlled for self-reported sex assigned at birth, age, ethnicity, education level, and country of residence. Descriptive demographic statistics were done using the “CrossTable” function from the “gmodels package” for Pearson's χ^2^ tests (for categorical and binary covariates) and the “wilcox.test” function from the “stats” package for the Mann–Whitney U tests (for continuous covariates). Missingness for the covariates of age, education level, ethnicity, and country of residence was addressed by creating five completed datasets (using predictive mean matching for five imputations) and pooling the results according to Rubin's rules using the “mice” and “pool” functions of the “MICE” package.^28^ To minimise type 1 errors from multiple testing, we used the false discovery rate correction and used a p threshold of 0·05 across all analyses.^29^

We ran a binomial logistic regression model controlling for all the covariates listed previously, as well as the interaction of sex and diagnosis for all outcomes in our main analyses. If there was a significant interaction, we used the “glht” function of the “multcomp” package (for the adjusted model) and sex-stratified Fisher's exact tests (for the unadjusted model) to estimate sex-specific values and reported the sex-specific results in place of the main models.

Regarding the qualitative analysis, we used NVivo version 12 (for the coding process) and R version 3.6.2. Using the frequency data from the content analysis, we did Fisher's exact tests to quantify the relative frequency of self-reporting aspects of recreational drug use among autistic and non-autistic individuals. To reduce risk of type 1 errors, we ran tests of significance to identify the relative frequency of discussing particular themes, rather than using individual codes to do so. We limited our analyses on subthemes to participants who reported information related to that theme, rather than on the entire sample. We used the false discovery rate correction and a p threshold of 0·05 across all analyses.

### Role of the funding source

The funders of the study had no role in study design, data collection, data analysis, data interpretation, or writing of the report.

## Results

Survey recruitment began on Feb 7, 2018, and ended on Aug 26, 2019; with two pauses in survey recruitment (from May 13, 2018, to June 28, 2018, and from April 8, 2019, to Aug 20, 2019) to consider different means of advertising the survey. No changes were made to the survey after these pauses. We did a sensitivity analysis covarying for each of the three time periods for adjusted regression analyses; there were no significant differences, as determined by z-tests and full results are provided in the appendix (p 4).

**Figure 1:**
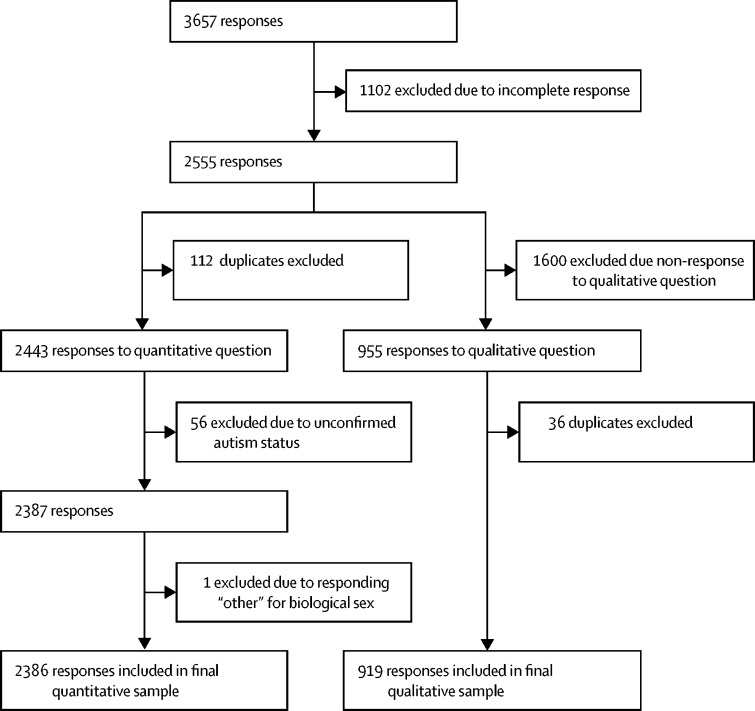
Study profile

At the end of the recruitment, 3657 individuals across 62 different countries accessed the survey; 1102 (30%) of the original participant responses were excluded due to incomplete responses, meaning that they withdrew from the survey before completing required questions related to their demographics (figure 1). 783 (71%) of these individuals withdrew from the survey before answering any survey questions. An additional 56 (2%) individuals were excluded from the quantitative sample due to unconfirmed autism diagnosis status (specifically, self-diagnosed as autistic, suspected autism, or were awaiting autism assessment). However, in the qualitative sample, these individuals were not excluded and were instead incorporated into the autistic (n=6) or non-autistic (n=23) cohorts on the basis of their own designation. One intersex participant was excluded from the quantitative sample, as our analysis strategy controlled for sex.

Both the samples for the quantitative and qualitative analyses were predominantly composed of female individuals, White individuals, UK residents, and those without intellectual disability (table 1). There were significant group differences, and these differences were expected based on the methodology and recruitment strategies used. There were no significant differences in age between the autistic and control groups; the mean age was 41·04 years (SD 14·41) in the autistic group and 41·86 years (15·59) in the control group of the quantitative sample. Data presented in table 1 are demographic data before imputation. The results remain highly similar after imputation. The mean ages of the qualitative sample were similar and can also be found in table 1.

**Table 1:** Participant demographics

		**Autistic group**	**Non-autistic group**	**Effect size**	**p values**
**Quantitative sample demographics (1183 autistic individuals, 1203 non-autistic individuals)**					
Age, years		41·04 (14·41)	41·86 (15·59)	0·019*	0·34
Age categories, years					
	16–29	303 (25·61%)	311 (25·85%)	..	..
	30–39	250 (21·13%)	240 (19·95%)	..	..
	40–49	252 (21·30%)	252 (20·95%)	..	..
	50–59	214 (18·09%)	206 (17·12%)	..	..
	60–69	113 (9·55%)	127 (10·56%)	..	..
	≥70	25 (2·11%)	52 (4·32%)	..	..
	Missing	26 (2·20%)	15 (1·25%)	..	..
Biological sex		..	..	0·058*	0·0045
	Female	746 (63·06%)	825 (68·58%)	..	..
	Male	437 (36·94%)	378 (31·42%)	..	..
Ethnicity		..	..	0·055*	0·0068
	White	1045 (88·33%)	1020 (84·78%)	..	..
	Non-White	135 (11·42%)	183 (15·21%)	..	..
	Missing	3 (0·25%)	0	..	..
Education		..	..	0·094†	<0·0001
	No formal qualifications	57 (4·82%)	14 (1·16%)	..	..
	Further vocational qualifications	215 (18·17%)	138 (11·47%)	..	..
	Secondary school or high school	211 (17·84%)	171 (14·21%)	..	..
	University undergraduate	354 (29·92%)	354 (29·43%)	..	..
	University postgraduate	344 (29·08%)	523 (43·47%)	..	..
	Missing	2 (0·17%)	3 (0·25%)	..	..
Country of residence		..	..	0·053*	<0·0001
	UK	842 (71·17%)	759 (63·09%)	..	..
	USA	120 (10·14%)	174 (14·46%)	..	..
	Germany	31 (2·62%)	33 (2·74%)	..	..
	Australia	33 (2·79%)	20 (1·66%)	..	..
	Other	156 (13·19%)	214 (17·79%)	..	..
	Missing	1 (0·08%)	3 (0·25%)	..	..
Intellectual disability		..	..	0·071*	0·00054
	Self-identified	21 (1·78%)	4 (0·33%)	..	..
**Qualitative sample demographics (429 autistic individuals, 490 non-autistic individuals)**					
Age, years		40·95 (14·42%)	41·81 (15·60%)	−0·047*	0·15
Age categories, years					
	16–29	76 (17·72%)	120 (24·49%)	..	..
	30–39	108 (25·17%)	114 (23·27%)	..	..
	40–49	128 (29·84%)	121 (24·69%)	..	..
	50–59	64 (14·92%)	77 (15·71%)	..	..
	60–69	35 (8·16%)	45 (9·18%)	..	..
	≥70	10 (2·33%)	8 (1·63%)	..	..
	Missing	8 (1·86%)	5 (1·02%)	..	..
Biological sex		..	..	0·047*	0·13
	Female	278 (64·80%)	291 (59·39%)	..	..
	Male	150 (34·97%)	199 (40·61%)	..	..
	Other	1 (0·23%)	0	..	..
Ethnicity		..	..	0·057*	0·083
	White	383 (89·28%)	421 (85·92%)	..	..
	Non-White	44 (10·26%)	69 (14·08%)	..	..
	Missing	2 (0·47%)	0	..	..
Education		..	..	0·10†	<0·0001
	No formal qualifications	15 (3·50%)	9 (1·84%)	..	..
	Further vocational qualifications	84 (19·58%)	59 (12·04%)	..	..
	Secondary school or high school	70 (16·32%)	47 (9·59%)	..	..
	University undergraduate	142 (33·10%)	150 (30·61%)	..	..
	University postgraduate	118 (27·51%)	225 (45·92%)	..	..
	Missing	0	0	..	..
Country of residence		..	..	0·060*	0·0099
	UK	300 (69·93%)	289 (58·98%)	..	..
	USA	49 (11·42%)	82 (16·73%)	..	..
	Germany	13 (3·03%)	13 (2·65%)	..	..
	Australia	8 (1·86%)	10 (2·04%)	..	..
	Other	59 (13·76%)	96 (19·59%)	..	..
	Missing	0	1 (<1%)	..	..

Data are mean (SD) or n (%). p-values and effect sizes were from Pearson's χ^2^ tests (for binary or categorical covariates) or from a Mann-Whitney U test (test of differences in means for continuous covariates). Effect sizes were based on measures of Phi (φ) for binary covariates or Cramér's V (V) for categorical covariates, or for measures of r (equal to Z statistic/✓[sample size]) for continuous covariates. All records with missing outcome data were excluded from the relevant test.

* Indicates a small effect.

† Indicates a small-to-medium effect.

Overall, we found some quantitative differences in alcohol use between autistic and non-autistic adolescents and adults. Compared with non-autistic individuals, autistic individuals were less likely to report regularly consuming alcohol (≥3 days per week on average; 16·0% of autistic individuals *vs* 22·2% of non-autistic individuals; adjusted model: odds ratio [OR] 0·69, 95% CI 0·55–0·86; p=0·0022) or engaging in binge-drinking (≥5 alcoholic beverages per average session; 3·8% *vs* 8·2%; adjusted model: OR 0·38, 0·26–0·56; p<0·0001; table 2). The binomial logistic regression analyses that included an additional interaction term for sex and diagnosis showed significant interactions for ever having smoked, smoking weekly or more, and ever having used recreational substances (table 2). Autistic male individuals were less likely than non-autistic male participants to report ever having smoked (50·8% of autistic male participants *vs* 64·6% of non-autistic male participants; adjusted OR 0·50; 95% CI 0·32–0·76; p=0·0022) or ever having engaged in recreational drug use (35·4% *vs* 52·7%; adjusted OR 0·53; 95% CI 0·35–0·80; p=0·0022). There were no significant differences between female groups (table 2). Bar graphs showing the full distribution for responses regarding alcohol use and smoking are provided in the appendix (pp 9–11).

**Table 2:** Substance use of autistic individuals compared with non-autistic individuals

	**Autistic group**			**Non-autistic group**			**Unadjusted model**		**Adjusted model**	
	Yes, n*	Total, n†	%	Yes, n*	Total, n†	%	Odds ratio (95% CI)	p values	Odds ratio (95% CI)	p values
Consumes alcohol ≥3 days per week	189	1182	15·99%	267	1202	22·21%	0·67 (0·54–0·82)	0·00043	0·69 (0·55–0·86)	0·0022
Consumes ≥5 alcoholic beverages per average session	45	1182	3·81%	98	1202	8·15%	0·45 (0·31–0·64)	<0·0001	0·38 (0·26–0·56)	<0·0001
Ever smoked (female individuals)	367	746	49·20%	447	824	54·25%	0·82 (0·67–1·00)‡	0·077	0·81 (0·60–1·09)§	0·47
Ever smoked (male individuals)	222	437	50·80%	244	378	64·55%	0·57 (0·43–0·75)‡	0·00036	0·50 (0·32–0·76)§	0·0022
Smoking weekly or more (female individuals)	236	746	31·64%	271	824	32·89%	0·94 (0·76–1·17)‡	0·67	0·93 (0·68–1·29)§	1·00
Smoking weekly or more (male individuals)	143	437	32·72%	146	378	38·62%	0·77 (0·58–1·03)‡	0·11	0·64 (0·41–1·00)§	0·077
Second-hand smoke exposure	384	1178	32·60%	336	1184	28·38%	1·22 (1·02–1·46)	0·052	1·19 (0·99–1·42)	0·10
Ever used drugs (female individuals)	280	745	37·58%	294	822	35·77%	1·08 (0·88–1·33)‡	0·55	1·11 (0·82–1·50)§	1·00
Ever used drugs (male individuals)	154	435	35·40%	199	378	52·65%	0·49 (0·37–0·65)‡	<0·0001	0·53 (0·35–0·80)§	0·0022

Unless otherwise specified, the unadjusted model refers to the outcome tested in the full population and the adjusted model refers to the binomial logistic regression analyses adjusted for age, biological sex, ethnicity, education, and country of residence.

* Number of respondents who designated each statement as true.

† Number of respondents who answered each question.

‡ Used a sex-stratified unadjusted model to estimate sex-specific odds ratios, CIs, and p values.

§ Adjusted model refers to sex-specific outputs of binomial logistic regression analyses adjusted for age, biological sex, interaction of sex and diagnosis, ethnicity, education, and country of residence.

**Figure 2:**
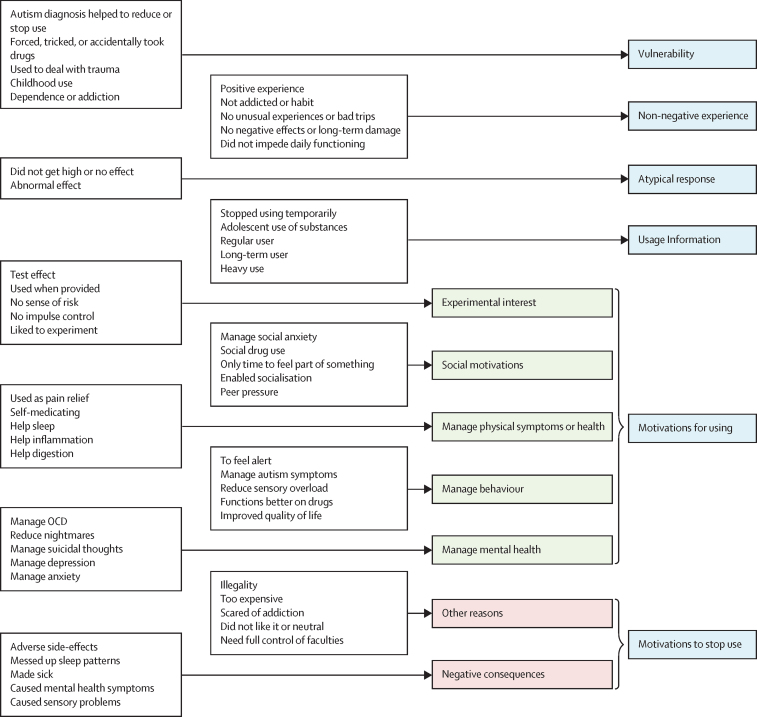
Selected codes, subthemes, and themes of substance use among autistic and non-autistic individuals The blue boxes represent the themes. The green boxes represent the subthemes for the theme called motivations for using. The red boxes represent the subthemes for the theme called motivations to stop use. The white boxes provide selected codes for each theme or subtheme; a list of all codes can be found in the appendix (p 1). OCD=obsessive-compulsive disorder.

Regarding our qualitative analyses, we identified 111 individual codes inductively that correspond to various aspects of the experience of substance use for 919 autistic and non-autistic individuals. Figure 2 provides selected codes and the themes (and subthemes for the “barriers” and “motivations for using” themes). A version of the figure that incorporates all codes into the structure can be found in the appendix (p 1). The panel provides selected quotes supporting each of the themes and subthemes identified.

Panel **Quotations supporting each theme and subtheme**

**Usage information**

“MDMA and derivates, around three years, occasionally. LSD, around three years, occasionally THC (marihuana), three years, quite regularly, though not daily. It was my first drug and a harmful one for me.” – Non-autistic male individual, aged 40–49 years

“in the past (up til 10 yrs ago): alcohol, daily, up until 9 yrs ago LSD, occasionally for a decade ether, a few weekends a year ketamine, occasionally heroin, daily for a couple years (ending 10yrs ago) cocaine, few times a week, up until 10yrs ago lots of pils, tons of pills...amphetamines, methadone, etc. (misc. pharms) recent: DXM daily up til last year. past and current: cannabis, daily for the past 25+yrs sugar, lots until recently. TONS. stuff i cant remember, of course.” – Autistic male individual, aged 40–49 years

**Non-negative experience**

“Smoked cannabis everyday for just over a decade between the ages of 19 to 33. Took small amounts of LSD (1 tab) every month or so for around two years, when I was 22–24. Occasionally took Ecstasy (maybe once every three months?) for around 5 years. Never experienced any ill effects.” – Non-autistic male individual, aged 40–49 years

“I am 33. I first took ecstasy in Jan 1999. I took it, because I was suicidal and I thought it would kill me. [...] it didn't kill me and I experienced happiness for the first time in my life…I consider myself to have been a functioning recreational drug addict between the ages of 14–28. I saw it as a point of pride that I partied harder than everyone else and still got up for school/uni/work. I have never missed anything or messed up anything in life through taking drugs, I am too intelligent and have too much self control to allow that to happen. The only reason I have friends is because I used to take drugs. I have no interest in other people and don't trust anyone, but ecstasy changes that. Going to Gatecrasher and being part of trance & hardhouse club culture was the best time of my life. I am glad that I got to have that experience as it was the only time I have ever been able to feel part of something…” – Autistic non-binary individual, aged 30–39 years

**Motivations for using: experimental interest**

“marijuana, about 1 year since it became legal in my state” – Autistic non-binary individual, aged 30–39 years

“Good few timemes amphetamines or ecstasy .. twice lsd/.. smoked daily joints good few years I didn't like the loss of short term memory, I stopped... I liked to experiment in 20s but I love reality and to be myself without any use of substances.” – Non-autistic female individual, aged 30–39 years

**Motivations for using: managing physical symptoms or health**

“…The amphetamine allows me to eat food as otherwise I cannot eat anything without extreme pain, discomfort would last up to 6 hour per day and the pain at least 4 hours. i lose all quality of life without amphetamine. i used to get it on prescription The Cannabis i have smoked for 3/4 years and only continue to do so because my Hospital told me i was curing my diseases” – Autistic female individual, aged 40–49 years

“Cannabis - I still use, I consume one hash truffle per evening to keep my pain levels under control, keep my mood level and help me sleep.” – Autistic non-binary individual, aged 40–49 years

“Cannabis: to try and treat seizures, approx 4 year, age 17–21…” – Non-autistic male individual, aged 30–39 years

“Cannabis - Occaisional recreational aged 18–25. For past 18 months - more regularly to control pain & muscle spasm”– Autistic female individual, aged 50–59 years

**Motivations for using: managing behavior**

“Cannabis - ongoing for mental health, sensory overload & pain relief” – Autistic female individual, aged 30–39 years

“…I used amphetamines approx twenty times over same time period. I found that this drug use alleviated my autistic symptoms enabled me to socialise/go out and reduced agoraphobia…” – Autistic female individual, aged 40–49 years

“…Sativa seems to alter my behavior in a way I become more sociable, stim less and am less prone to meltdowns.” – Autistic individual, aged 20–29 years

“marijuana, speed, MDMA, cocaine, mephedrone - used over a few years during adolescence/ early 20s mainly and mostly amphetamines used to feel alert and more ‘normal' rather than to get high…” – Autistic non-binary individual, aged 30–39 years

“…Out of all the drugs my favourite was ecstasy it got me doing things, thinking clearly and I could chat to people. I also appreciated the effects mushrooms had on my synesthesia.” – Autistic transgender male individual, aged 30–39 years

**Atypical response**

“…I no longer try any recreational drugs since I seem to have abnormal responses to them.” –Autistic female individual, aged 30–39 years

“…No one suspects me of being “high”, I don't get the munchies, or talk endlessly. Instead of watching every muscle move in people's faces and frantically trying to process whether I am in trouble or not, I can just work and be okay. I tried cocaine in the 1970s but it did nothing and I didn't like being around the people who used it…” – Autistic female individual, aged 60–69 years

**Motivations for using: managing mental health**

“…Weed/hash - Currently. Helping better to deal with anxiety/depression than all the medication I've been diagnose so far. I don't get high, but gives me a sense of calm and control of myself…” – Non-autistic male individual, aged 30–39 years

“…I've recently started to use small amounts of pure weed again, been using and significantly lower the dosage of the other antidepressant I used. It has drastically improved my life, as both antidepressants were not without side effects. (Weight gain, extreme sweating, significantly lowered libido & almost unable to orgasm)”– Autistic male individual, aged 30–39 years

“Cannabis it helped with the panic attacks and nightmares, i also stopped smoking everything on 28 dec 2017 not had anything since then” – Autistic male individual, aged 40–49 years

“…I've been on mild opiates for various chronic pain conditions for several years, but find they help my autistic depression and rage issues, sleep and PTSD as well. It's an open question as to whether that could be called “recreational,” however I consider it legitimately medical, but it's worth mentioning. Especially as I think opiates are far too demonised and under-explored, considering how incredibly helpful they are for autism specifically–autism is like being born with PTSD because you're triggered by everything and constantly anxious, but opiates smooth that out and make your brain feel “normal”. I've never got similar help from any antidepressants/neuroleptics, and the side effects of the latter have been far more devastating for my health than opiates ever have been, causing permanent physical damage. Opiates, on the other hand, have improved my mental and physical health dramatically (leading me to suspect that whatever it was I was born with is to do with some kind of dysregulation of the opiate receptors).”Autistic female individual, aged 30–39 years

**Motivations to stop use: negative consequences**

“…i started smoking when i was 18, and stopped smoking regularly when i was 24. i stopped because it just stopped being fun. i found that it began to make me quite depressed...” – Autistic male individual, aged 40–49 years

“Cannabis for 3 years. Several daysper week for 2 years. I stopped because it gave paranoide symptomes including delusions.” – Non-autistic female individual, aged 30–39 years

“Tried cannabis once when younger. Wasn't for me. Gave me severe anxiety.” – Autistic female individual, aged 30–39 years

“Lsd a few times in high school and pot. Lsd made me absolutely pain free but made me yak, all pot gives me severe migraines. No drugs since 1992.” – Autistic non-binary individual, aged 40–49 years

**Motivations for using: social motivations**

“marijuana - probably used on and off for 3 years once or twice a week in my late teens/ early 20's (not enjoyable made me throw up) - did it due to peer pressure. Had one 24 hour psychosis when I ate a large amount instead of smoking it. amphetamines - once (felt very unpleasant agitation)…” – Autistic female individual, aged 60–69 years

“…Used infrequently (couple of times per year), and only at parties or clubs since then (ongoing). Often in combination with each other or alcohol…” – Autistic male individual, aged 40–49 years

“I haven't used drugs for years. I used to use MDMA/ecstasy pills about once or twice a month - I found it really helped me socially. I used speed occasionally - this turned me into an outgoing extrovert that could talk to anyone (the polar opposite of what I'm usually like). I used cocaine and cannabis occasionally (I didn't enjoy smoking cannabis and it didn't have a pleasant effect on me, I only did it because of social pressures). I tried ketamine and it made me very ill. I hardly ever drink alcohol now because my body can't tolerate it these days. I used to drink a lot when I was younger and it helped me socially at lot but it also used to make me feel very depressed as it was wearing off...” – Autistic female individual, aged 30–39 years

**Vulnerability: substance misuse**

“Marihuana, several grams a Day for 2 maybe 3 years. Became addicted. Had help from addiction care, both outpatient and clinical” – Autistic female individual, aged 40–49 years

“I got drunk every day for 20 years. It helped me cope. I stopped drinking in 2011 and couldn't cope. I was sent to psychiatrist and told I was autistic” – Autistic male individual, aged 50–59 years

“I first took ecstasy in Jan 1999. I took it, because I was suicidal and I thought it would kill me. Unfortunately, it didn't kill me and I experienced happiness for the first time in my life…I consider myself to have been a functioning recreational drug addict between the ages of 14–28. I saw it as a point of pride that I partied harder than everyone else and still got up for school/uni/work…There were many times when I tried to stop taking drugs, but I always ended up back in the same place taking them. The only reason I stopped was getting diagnosed with Asperger's and finally getting psychological help when I was 28. If I had easy and safe access to opiates I would start taking them. I no longer have interest in drugs which speed things up, I would like something which knocks me out and makes me forget about how much I hate my life.”– Autistic non-binary individual, aged 30–39 years

**Motivations to stop use: other reasons**

“Cannabis, intermittently. Most years not at all. But if I could get it on nhs would help me with my sleep pattern” – Autistic male individual, aged 20–29 years

“…But I don't do it much anymore as money is a problem so I just put up with feeling like crap.” – Autistic male individual, age unknown“…opium - about 3 times (fantastic but was too scared of addiction to continue, plus it wasn't something that easy to get hold of)” – Autistic female individual, aged 60–69 years

**Vulnerability**

“lsd, mdma, mushrooms, ketamine found them to be integral in processing previous long term abuse and preparing (unintentionally) for therapy, and giving me a reason to live in a bad time in my life” – Autistic female individual, aged 30–39 years

“It's part of my shameful past, but desomorphine, amphetamine(s)[uncertain which] and I've sniffed glue... Over a period of about 2 years, between the ages of 8 and 10, on several occasions. I couldn't say how often exactly. It was sometimes forced upon me. As of then, never again. I never wanted it, but I can't deny that I did do it...” – Autistic male individual, aged 16–19 years

“…I tried speed once by mistake on holiday in Corfu when a Club Rep offered me a vitamin unfortunately I believed him (age 20). I tried Cocaine once (age 33)…” – Autistic female individual, age unknown“…Soon cannabis became my best screen to hide behind. I have lived here ever since. I married an Englishman in 1971, then children and teaching happily put a stop to my bohemian life. In the 90s I went back to cannabis, it felt calming and scattered my suicidal thoughts. My depression can become major, I made serious attempts in the past. I seriously regret the pain I caused to my children. At present, and for a number of years now, I have been smoking it daily and relay on it as if it were a trusted friend. It works at keeping me going, I say, ‘to stay alive'. Since 1978 I have had psychiatric treatment with all sorts of meds, CAT, CBT and DBT therapy, even 3 courses of ECT and different diagnoses that didn't “fit” and all helped marginally. I am still on medication but totally resigned that depression is here to stay. After some 12 years of asking to be given an AS assessment only to be met with rejection and humiliation, I was even told I was choosing an easy way out to avoid the responsibility of changing my pattern of life, in 2015 I was finally diagnosed as an Asperger's. It “fits” and has helped me to understand most of my life and difficulties....unfortunately there is hardly any support out there for adults, very few understand, even medics, or make allowances for people of my age. I perceive this as saying, “You got here so far, get on with it then. You must know what to do by now.' Hence cannabis as a trusted comforter at 67.” – Autistic female individual, aged 60–69 years

Grammar or typographical errors were not corrected; specific names of prescribed medications have been omitted to protect anonymity of participants.

We also identified the frequency of all codes for autistic and non-autistic individuals separately (appendix pp 2–4). Autistic individuals were less likely than non-autistic individuals to provide any qualitative information about their substance use (OR 0·83, 95% CI 0·70–0·98; p=0·045). Autistic individuals were more likely to report an atypical response to drugs (regarding their experience or tolerance, or both; OR 6·56, 2·20–26·38; p=0·00027), as well as experiences of substance use that indicate vulnerability (OR 4·16, 1·90–10·05; p=0·00027), including being forced or tricked to use substances, childhood use of substances, suicidality, trauma, and addiction or dependence (table 3).

**Table 3:** Relatively increased likelihood of reporting specific themes and subthemes related to substance use by autistic adults compared with non-autistic adults

	**Autistic group, n (%)**	**Non-autistic group, n (%)**	**Odds ratio (95% CI)**	**p values**
Frequency of reporting themes (429 autistic individuals, 490 non-autistic individuals)*				
Atypical response	22 (5·13%)	4 (0·82%)	6·56 (2·20–26·38)	0·00027
Motivations for using	91 (21·21%)	47 (9·59%)	2·54 (1·71–3·79)	<0·0001
Motivations to stop use	58 (13·52%)	27 (5·51%)	2·68 (1·63–4·49)	0·00023
Non-negative experience	11 (2·56%)	6 (1·22%)	2·12 (0·71–7·05)	0·21
Usage information	427 (99·53%)	488 (99·59%)	0·88 (0·06–12·12)	1·00
Vulnerability	31 (6·99%)	9 (1·84%)	4·16 (1·90–10·05)	0·00027
**Frequency of specific motivations for using (91 autistic individuals, 47 non-autistic individuals)**†				
Experimental interest	4 (4·40%)	2 (4·26%)	1·03 (0·14–11·85)	1·00
Social motivations	36 (39·56%)	31 (65·96%)	0·34 (0·15–0·75)	0·0095
Manage physical symptoms or health	39 (42·86%)	12 (25·53%)	2·18 (0·95–5·23)	0·097
Manage mental health symptoms	32 (35·16%)	7 (14·89%)	3·08 (1·18–9·08)	0·032
Manage behaviour	26 (28·57%)	2 (4·26%)	8·89 (2·05–81·12)	0·0017
**Frequency of specific motivations to stop use (58 autistic individuals, 27 non-autistic individuals)**†				
Negative consequences	25 (43·10%)	12 (44·44%)	0·95 (0·34–2·65)	1·00
Other reasons	43 (74·14%)	21 (77·78%)	0·82 (0·23–2·66)	1·00

‡These analyses provide results from Fisher's exact tests in which the total sample includes only participants who noted a specific motivation to stop using substances.

* These analyses provide results from Fisher's exact tests in which the total sample includes all participants who provided any qualitative data.

† These analyses provide results from Fisher's exact tests in which the total sample includes only participants who noted a specific motivation for using substances.

Among those who reported a specific motivation for using drugs, autistic individuals were more likely to report using substances to manage mental health symptoms and behaviour, but were less likely to report social motivations for drug use (table 3). Among participants who reported a specific motivation for drug use, autistic individuals were nearly nine times more likely to report using recreational substances to manage behaviour than non-autistic individuals (OR 8·89; 95% CI 2·05–81·12; p=0·0017) and over three times more likely to reporting using recreational substances to manage mental health symptoms than were non-autistic individuals (OR 3·08; 95% CI 1·18–9·08; p=0·032**)**. Autistic individuals also reported using substances as a form of self-medication for physical health symptoms (including sleep, digestion or eating, and pain) but the frequency of this reporting was not different from that of non-autistic individuals. Five individuals specifically reported that receiving their autism diagnosis was relevant to reducing or ending their substance use.

## Discussion

Autistic individuals were less likely to report consuming alcohol regularly or binge-drinking than non-autistic individuals; however, our mixed-methods approach revealed possible points of concern regarding the substance use of autistic individuals, including wide-ranging sex differences and qualitative differences in motivations for drug use. Autistic male individuals were far less likely to report ever having smoked or having ever used recreational substances than non-autistic male individuals, whereas there were no significant differences between these patterns for autistic female individuals. To our knowledge, this finding is the first evidence that autistic male individuals are particularly unlikely to engage in substance use compared with non-autistic male individuals; it might also suggest that the sex-specific pattern of substance use in the general population (eg, male individuals are more likely than female individuals to engage in substance use) might be different to that of autistic individuals,^24^ although two previous studies showed small or non-significant sex differences.^14^, ^19^ Cross-sectional convenience samples (as used in the present study) provide a unique opportunity for recruitment of large samples of diagnosed autistic female individuals,^3^, ^4^, ^5^, ^6^, ^7^ without which analyses on sex differences would not be possible. This work emphasises the importance of recruiting autistic female individuals in research, as they might have unique risk factors and are now widely reported to have worse outcomes regarding physical health, mental health, and mortality.^14^, ^22^, ^30^

Among those who provided any information regarding their motivations for using drugs, autistic individuals were nearly nine times more likely than non-autistic individuals to report using them to manage behaviour specifically. Although the terms masking, compensation, and camouflaging were not directly used by participants and might not apply to all instances of descriptions of managing behaviour, the descriptions provided frequently correspond to existing literature and definitions of these concepts;^4^, ^5^, ^6^, ^7^, ^8^ individuals described using drugs to eliminate, control, or reduce autism or symptoms of autism (eg, sensory overload, stimming behaviour, improving overall function, improving perception) and other comorbid symptoms (eg, ADHD). One autistic individual noted “I smoke pot to make my anxiety and autism go away. It's the only time I fell on the same wave length as everyone else”). Although causal links have not yet been established, compensation and camouflaging have been linked to high rates of mental health conditions and increased risk of suicidality.^4^, ^5^, ^8^, ^9^ Future research should investigate the role of compensatory and camouflaging strategies in motivating substance use of autistic individuals more specifically.

Autistic individuals reported using substances as a form of self-medication for both mental and physical health symptoms, although only mental health symptoms showed a significant increase in reporting among autistic compared with non-autistic individuals. Many individuals did not view this self-medication as negative, instead indicating that use of marijuana (or more rarely other substances) provided them with a higher quality of life, as proposed previously by the self-medication hypothesis.^18^, ^31^ Understanding the complex relationships among substance use, physical health, and mental health is essential, as several studies now indicate that autistic individuals are at increased risk of a wide variety of chronic physical and mental health conditions,^12^, ^13^, ^14^, ^16^, ^20^, ^22^ and substance use can have deleterious effects on physical and mental health.^21^ A previous study from our research group at the Autism Research Centre, University of Cambridge, UK, has shown that alcohol use and smoking do not fully explain differences in prevalence of physical health conditions between autistic and non-autistic individuals;^22^ yet, it is possible that substance use operates in a positive feedback loop by worsening physical and mental health conditions.

This study bolsters previous findings by supporting greater likelihood of self-reported vulnerability associated with substance use among autistic compared with non-autistic individuals.^32^, ^33^ Five autistic participants in this study specifically noted that their autism diagnosis was crucial to discontinuing their use or misuse of substances, emphasising the importance of timely diagnosis of autism. In addition, we have identified new areas of risk among autistic individuals, including forced or accidental use of drugs and childhood use of drugs. This study provides preliminary evidence that substance use or misuse might have a complex association with vulnerability, with substances being used both to cope with symptoms (eg, to deal with trauma, suicidality) and to serve as a vehicle to exacerbate other forms of vulnerability (eg, eating disorders).

Previous qualitative studies have suggested that autistic adults might be motivated to use substances for the reasons such as compensatory or camouflaging strategies, mental health, physical health, and adverse life events or vulnerability.^18^, ^23^ However, our findings are, to the best of our knowledge, the first to clarify that autistic adults were far more likely than non-autistic adults to report substance use for these reasons. These findings have clinical implications. First, adverse life events, autism symptoms causing difficulty, mental health symptoms, and physical health symptoms might all serve as possible risk factors for substance use among autistic individuals. Second, autistic individuals might not be receiving appropriate management of behavioural, physical, or mental health symptoms from medical providers. Third, marijuana and other substances currently used for recreational drug use should be investigated as possible medical interventions for managing physical and mental health symptoms frequently comorbid to autism. Fourth, unwanted symptoms of autism, mental health, and physical health conditions (identified by patients themselves) might serve as key targets for intervention for reducing substance use. Fifth, approaching sensitive topics (eg, substance use) is essential to ensure appropriate safeguarding, particularly in light of evidence that substance use might be associated with risk of vulnerability among autistic individuals. As differences with social communication are a core feature of autism, autistic individuals have previously endorsed that taking extra time within appointments and honouring alternative forms of communication (such as written or online communication) might improve patient–provider communication;^34^ however, the efficacy of these strategies has not been tested directly.

Although the study includes a large sample of qualitative responses, several limitations should be noted. First, the study might be subject to the so-called winner's curse, meaning that it might include artificially inflated point estimates for group differences. Second, our study is subject to sampling and recruitment biases, as advertisements were circulated via social media, autism charities and support groups, and two networks of autistic individuals. As such, the non-autistic sample might be biased towards individuals with high autistic traits, an interest in autism, or undiagnosed autism; therefore, our results might underestimate true group differences between autistic and non-autistic individuals. Third, our sample primarily includes autistic individuals who are White, who do not have comorbid intellectual disability, and who have completed high school or higher education; therefore, the findings are probably not representative of the experiences of substance use of all autistic individuals. Fourth, we did not measure levels of autistic traits among formally diagnosed autistic individuals; although individuals would need to meet a threshold of autistic traits to receive a formal diagnosis of autism, we cannot be certain that our sex-specific groups have similar levels of autistic traits or symptoms, which is a clear limitation of our analyses testing sex differences in substance use. Fifth, our quantitative analyses are based on binary measures of substance use (some of which have been simplified from categorical reports), to provide the most clinically relevant information, which will have resulted in a loss of some power and information. We have also provided the unadjusted distributions of each original categorical variable (appendix pp 9–11). Sixth, our qualitative analysis is based on responses to a single open-ended question, which might not fully reflect all the attitudes, motivations, and experiences of autistic or non-autistic individuals across the adult lifespan regarding their substance use. Finally, the effect sizes and frequency data presented in relation to our qualitative analyses should be interpreted not as providing information about the actual prevalence of these experiences, but instead about the relative likelihood of autistic and non-autistic individuals reporting these themes in an unprompted fashion. A key limitation of this strategy is that autistic and non-autistic individuals might be differentially likely to report information in an unprompted fashion, owing to differences in communication style. To mitigate this risk where possible, reported group differences in specific subthemes were tested only among individuals who discussed the overarching theme (eg, we compared frequency of reported drug use to manage behaviour only among participants who discussed any specific motivation for using drugs).

The themes and subthemes identified by autistic individuals have clear clinical relevance, particularly with regards to the evidence of vulnerability and the evidence about unmet needs for managing behavioural, mental health, and physical health symptoms. To the best of our knowledge, this study provides the largest sample of qualitative data of its kind, and it newly identified and supports several themes and subthemes related to the experiences of substance use of autistic individuals without intellectual disability across the adolescent and adult lifespan (aged 16–90 years). On the basis of the results of the present study, clinicians should be aware of possible vulnerability related to substance use (including being forced or tricked to use substances, childhood use of substances, suicidality, trauma, and addiction or dependence) and should work cooperatively with patients to provide effective means of managing autistic patients' behavioural, mental health, and physical health symptoms.

## Data sharing

We can provide group-level data but not the underlying material itself, as our participants did not consent to having their data shared publicly. Underlying anonymised data will be stored until 5 years after the study ends and will only be made available to potential collaborators with ethics approval, after they submit a research proposal to the Autism Research Centre, University of Cambridge, UK.

## Declaration of interests

We declare no competing interests.
