# Supplementary material for: Understanding the substance use of autistic adolescents and adults: a mixed-methods approach
Source: Lancet Psychiatry. 2021 Aug;8(8):673–85. doi: 10.1016/S2215-0366(21)00160-7 (PMC8310943; doi:10.1016/S2215-0366(21)00160-7)
Supplement: Supplementary appendix [file mmc1.pdf]

THE LANCET
Psychiatry

Supplementary appendix

This appendix formed part of the original submission and has been peer reviewed. We post it as supplied by the authors.

Supplement to: Weir E, Allison C, Baron-Cohen S. Understanding the substance use of autistic adolescents and adults: a mixed-methods approach. *Lancet Psychiatry* 2021; published online July 1. http://dx.doi.org/10.1016/S2215-0366(21)00160-7.

Appendix

Supplementary Table 1 displays the frequency at which the autistic and non-autistic respondents to the survey for each code. It should be noted that this study provides information about the issues that participants chose to provide information, which may or may not correspond to true differences in behaviour (e.g. autistic individuals may be more likely to report that they use drugs to manage physical health problems but this does not mean that they are necessarily more likely do so than non-autistic individuals). This study provides more information about how autistic and non-autistic individuals feel about their substance use, what they view as important regarding their substance use, and provides some information about why individuals choose to use or not to use substances.

Supplementary Figure S1: Codes, Sub-Themes, and Themes of Substance Use of Autistic and Non-Autistic Adults

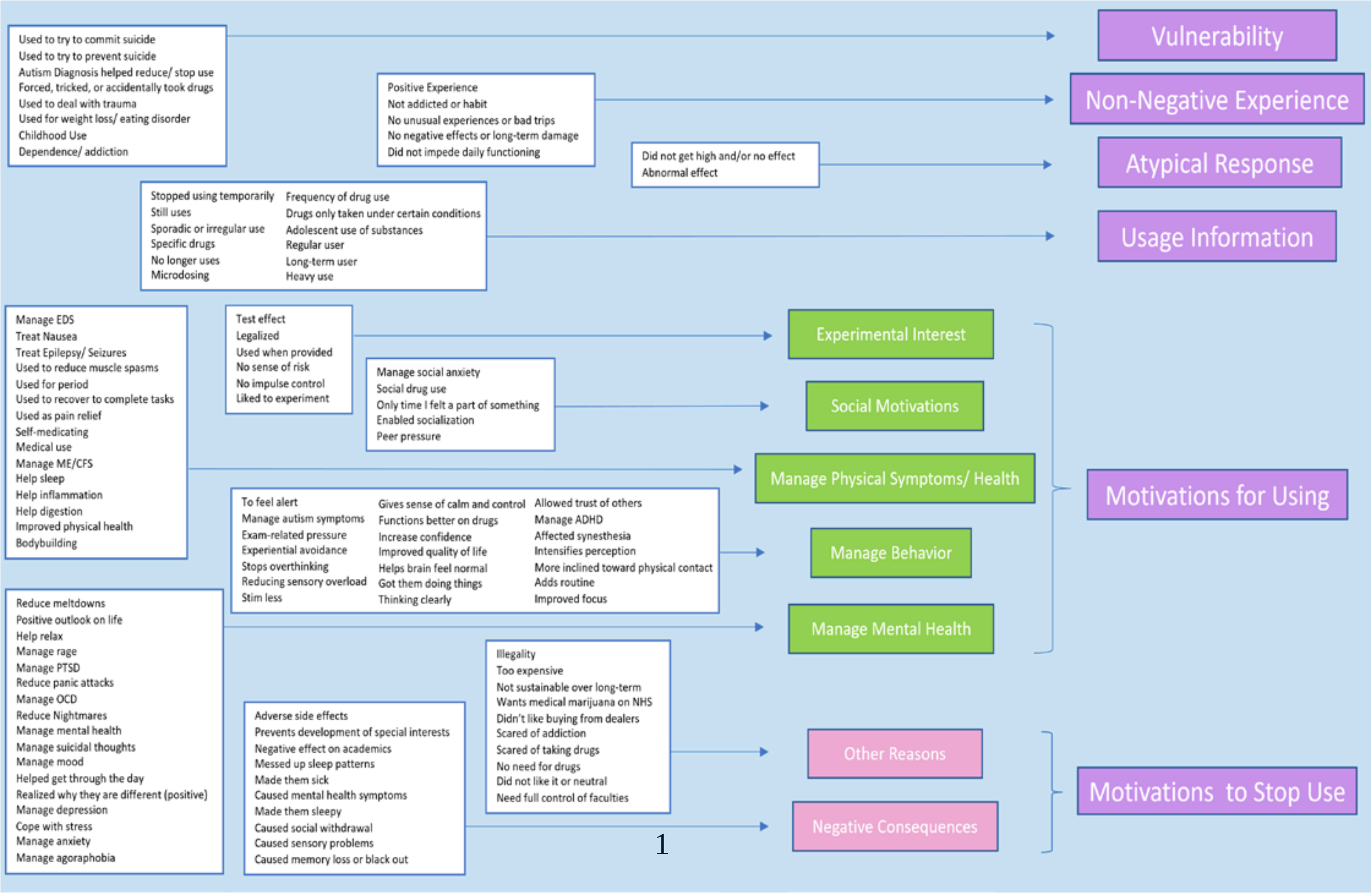

Supplementary Table S1: Frequency Table of Qualitative Responses for Each Code

	Autistic (n = 429)	Non-Autistic (n = 490)	Total (n = 919)
Drugs used for weight loss or eating disorder	2	0	2
Used Drugs to Try to Commit Suicide	1	0	1
Used Drugs to Try to Deal with Suicidality	2	1	3
Autism Diagnosis Helped Reduce/ Stop Use	5	0	5
Forced, tricked, or accidentally took drugs	6	3	9
Used Drugs to Deal with Trauma	3	0	3
Childhood Use of Substances	5	2	7
Dependence/ Addiction	15	5	20
Heavy Use	10	6	16
Regular User	99	106	205
Long term User	192	211	403
Stopped Using Drugs Temporarily	29	27	56
Still Uses	54	53	107
Sporadic or Irregular Use	245	319	564
Specific Drugs	426	482	908
No Longer Uses Drugs	178	165	343
Microdosing	2	4	6
Frequency of Drug Use	305	366	671
Drugs only taken under certain conditions	7	6	13
Adolescent use of substances	71	69	140
Positive Experience	1	2	3
Not Addicted or Habit	7	3	10
No Unusual Experiences or Bad Trips	1	0	1
No Negative Effects or Long term Damage	3	1	4
Did Not Impede Daily Functioning	2	1	3
Prevented Development of Special Interests	1	0	1
Negative Effect on Academics	3	0	3
Messed Up Sleep Patterns	1	0	1
Made Them Sick	3	0	3
Made Them Sleepy	0	1	1
Caused Social Withdrawal	1	0	1
Caused Sensory Problems	1	0	1
Caused Mental Health Problems/ Symptoms	12	4	16
Caused Memory Loss or Black Out	2	2	4
Adverse Side Effects	12	7	19
Did not like it or neutral	32	20	52
No Need for Drugs	2	1	3
Need Full Control of Faculties	1	0	1
Illegality	1	0	1
Too Expensive	2	0	2
Not Sustainable over long term	1	0	1
Wants medical marijuana on NHS	1	0	1
Didn’t like buying from dealers	1	0	1
Scared of Addiction	1	0	1
Scared of Taking Drugs	1	0	1
Manage Social Anxiety	2	0	2
Social Drug Use	25	31	56
Only Time I Have Felt a Part of Something	2	0	2
Drugs Enabled Them to Socialize	8	0	8
Peer Pressure	6	1	7
Manage EDS	1	0	1

Treat Nausea	1	1	2
Treat Epilepsy/ Seizures	0	1	1
Used to Reduce Muscle Spasms	1	0	1
Use for Period	1	0	1
Used to Recover to get a prescription	2	0	2
Used as Pain Relief	16	3	19
Self-medicating	11	5	16
Medical Use	3	0	3
Manage ME/CFS	0	1	1
Help Sleep	14	4	18
Help Inflammation	1	0	1
Help Digestion	1	0	1
Improved Physical Health	1	0	1
Bodybuilding	0	1	1
Reduce Meltdowns	1	0	1
Positive Outlook on Life	0	1	1
Help Relax	6	2	8
Manage Rage	1	0	1
Manage PTSD	1	0	1
Reduce Panic Attacks	1	0	1
Manage OCD	1	0	1
Reduce Nightmares	1	0	1
Manage Mental Health	1	1	2
Manage Suicidal Thoughts	1	0	1
Manage Mood	1	0	1
Helped Get Through the Day	3	0	3
Helped Realize Why They Were Different	1	1	2
Manage Depression	5	1	6
Cope with Stress	4	1	5
Manage Anxiety	8	3	11
Manage Agoraphobia	1	0	1
To Feel Alert	1	0	1
Manage Autism Symptoms	6	0	6
Exam-Related Pressure	1	0	1
Experiential Avoidance	1	0	1
Stops Overthinking	1	0	1
Reducing Sensory Overload	5	0	5
Gives Sense of Calm and Control	3	1	4
Functions Better on Drugs	2	0	2
Increase Confidence	1	0	1
Improved Quality of Life	3	0	3
Helps Brain Feel Normal	3	0	3
Got Them Doing Things	1	0	1
Thinking Clearly	1	0	1
Improved Focus	3	0	3
Allowed Trust of Others	1	0	1
Manage ADHD	1	0	1
Adds Routine	1	0	1
Stim less	1	0	1
Affected Synesthesia	1	0	1
Intensifies Perception	2	1	3
More Inclined toward physical contact	1	0	1
Test Effect	1	0	1
Legalized	1	0	1
Used When Provided	0	1	1
No sense of Risk	1	0	1

No impulse control	1	0	1
Liked to experiment	1	1	2
Did not get high and/or no effect	17	3	20
Abnormal Effect	8	1	9

Supplementary Table S2: Sensitivity Analysis for Time Point

	ß1	SE1	ß2	SE2	Z-test p-value
Consumes Alcohol 3+ Days per Week	-0.37	0.11	-0.35	0.12	0.90
Consumes 5+ Alcoholic Beverages per Session	-0.96	0.19	-0.94	0.20	0.99
Ever Smoked (Females)	-0.21	0.10	-0.24	0.11	0.86
Ever Smoked (Males)	-0.70	0.15	-0.74	0.15	0.85
Smoking Weekly or More when Smoking Most (Females)	-0.07	0.11	-0.07	0.12	0.99
Smoking Weekly or More when Smoking Most (Males)	-0.44	0.15	-0.45	0.16	0.99
Secondhand Smoke Exposure	0.17	0.09	0.19	0.10	0.90
Ever Used Drugs (Females)	0.10	0.11	0.10	0.11	0.98
Ever Used Drugs (Males)	-0.64	0.15	-0.63	0.15	0.93

ß1 and SE1 refer to the original model (covarying for age, biological sex, ethnicity, education, and country of residence, as well as interaction of sex and diagnosis where appropriate); ß2 and SE2 refer to the model which includes Time period as an additional covariate; z-test p-value provides the likelihood that the two models are significantly different

Note: values shown are rounded

ß = regression coefficient

SE = standard error

Supplementary Table S3: Ethnic Breakdown of Non-White Participants for Quantitative and Qualitative Samples

Quantitative Sample				
	Autistic (n = 1,183)		Non-Autistic (n = 1,203)	
	Number of Participants	Percentage of Autistic Sample (%)	Number of Participants	Percentage of Non-Autistic Sample (%)
Mixed Race	77	6.51	73	6.07
Asian	18	1.52	43	3.57
Latin American/ Hispanic	7	0.59	23	1.91
Arab/ Middle Eastern	0	0	17	1.41
Jewish	16	1.35	17	1.41
African/ Black/ Caribbean	6	0.51	9	0.75
Other	11	0.93	1	0.08
**Qualitative Sample**				
	Autistic (n = 429)		Non-Autistic (n = 490)	
	Number of Participants	Percentage of Autistic Sample (%)	Number of Participants	Percentage of Non-Autistic Sample (%)
Mixed Race	23	5.36	33	6.73
Asian	3	0.70	10	2.04
Latin American/ Hispanic	1	0.23	8	1.63
Arab/ Middle Eastern	0	0	2	0.41
Jewish	5	1.17	9	1.84
African/ Black/ Caribbean	3	0.70	1	0.20
Other	9	2.10	6	1.22

Specific Questions

We have included images of the questions relevant to the substance of autistic adults, in order to display the questions as they would have been presented to participants in the survey. As some of the questions may have been sensitive in nature to certain participants, all questions in this section were optional and participants were notified this at the beginning of this section of the survey.

In Figures S1 and S2 we asked participants to disclose information about their current alcohol use, including information on average frequency of alcohol use (Figure S1) as well as average quantity of units per day (Figure S2).

Figure S2: Frequency of Current Alcohol Consumption

In a typical week, how many days per week do you consume alcoholic beverages?

- ○ I do not consume alcoholic beverages
- ○ 1-2 days per week
- ○ 3-5 days per week
- ○ 6-7 days per week

Figure S3: Number of Drinks Currently Consumed per Day

On average, how many alcoholic beverages do you have on a typical day while drinking?

- ○ 1-2 alcoholic beverages
- ○ 3-4 alcoholic beverages
- ○ 5-6 alcoholic beverages
- ○ 7-8 alcoholic beverages
- ○ More than 8 alcoholic beverages

We also attempted to quantify participants' smoking behaviours or exposure. First, we asked if the participants had ever smoked (Figure S3). Second, if the participant replied 'Yes' to this question, they were shown an additional follow-up question about the frequency of their smoking when smoking the most (Figure S4). It should be noted that participants were shown an additional question about the quantity of cigarettes smoked; however, there was an error in the phrasing of

this question, and we did not analyse any of the results from this question. Third, we asked participants to disclose whether participants have exposure to second-hand smoke (Figure S5).

Figure S4: Whether or Not the Participant has Ever Smoked

Have you ever smoked a cigarette?

- ○ Yes
- ○ No

Figure S5: Smoking Frequency When Smoking the Most

When you were smoking most frequently, how often did you smoke cigarettes?

- ○ Daily
- ○ Weekly
- ○ Monthly
- ○ I have never smoked regularly

Figure S6: Second-hand Smoke Exposure

Do you have any exposure to second hand smoke?

- ○ Yes
- ○ No

In our final set of questions about substance use, we inquired about participants' use of recreational substances/ drugs. As with the questions regarding smoking, we first asked participants to provide information about whether they had ever used recreational substances (Figure S6). Finally, we included an open text, free-response question where we asked participants to provide any further information that they wished about the types of drugs used, their frequency of use, or their experiences (Figure S7). Although we received various levels of detail regarding drug use (making this question unsuitable for quantitative analysis), this question was used in our qualitative analysis.

Figure S7: Whether or Not the Participant has Ever Used Recreational Drugs

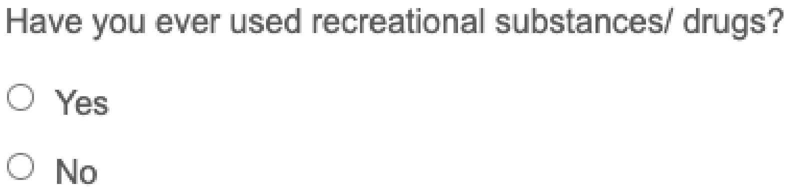
Have you ever used recreational substances/ drugs?

Yes

No

Figure S8: Free-text Question about Recreational Drug Use

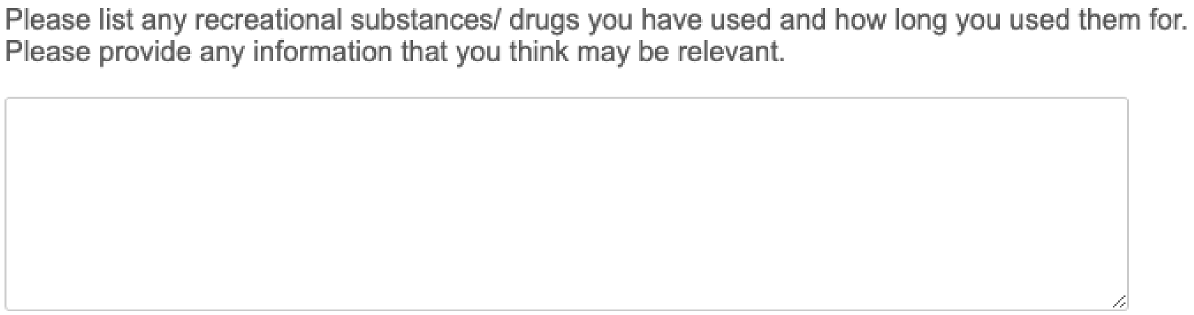
Please list any recreational substances/ drugs you have used and how long you used them for.
Please provide any information that you think may be relevant.

The main analyses included in the manuscript are binary representations of alcohol use and smoking, specifically targeted at determining whether or not autistic adults are more likely to engage in possible substance misuse compared to others. In addition, we have included bar graphs showing the full distribution of responses for both autistic and non-autistic adults for each of the questions (as originally asked) in Supplementary Figures S8-10 below.

Figure S9: Average Days Per Week of Alcohol Consumption of Autistic and Non-Autistic Adults

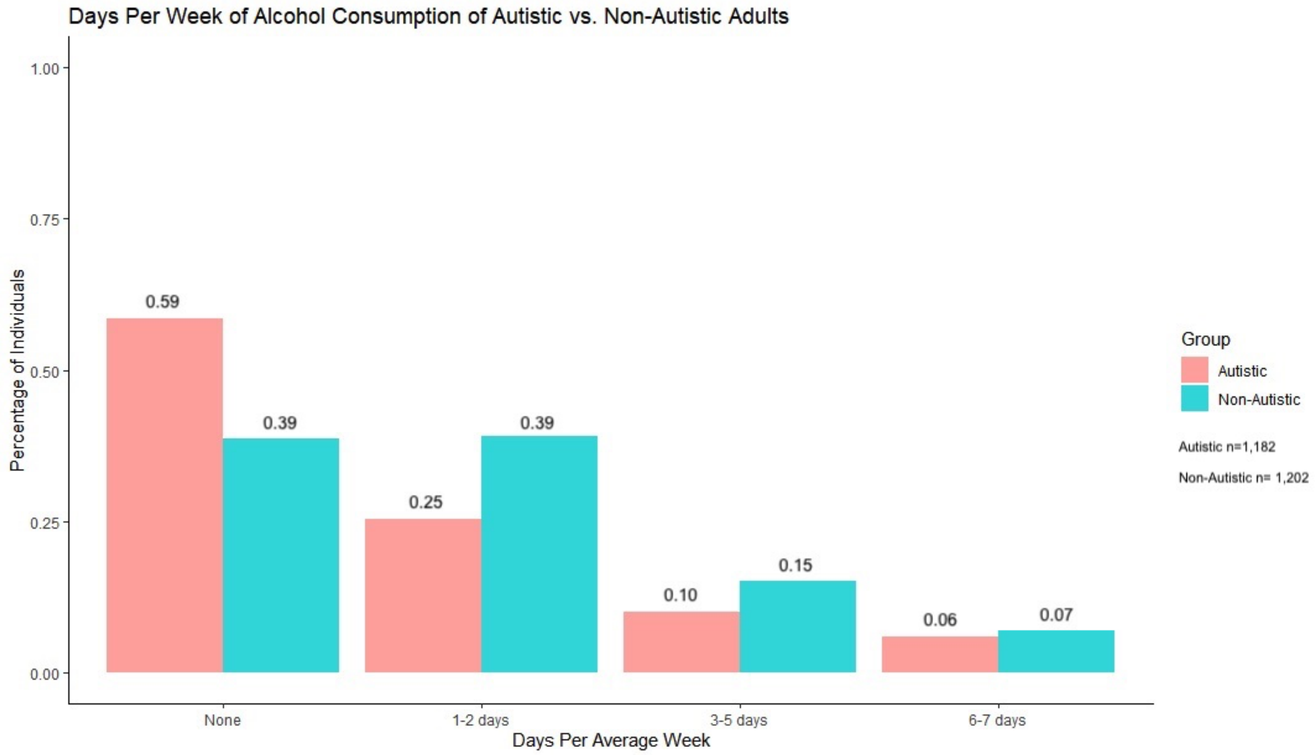

Figure S10: Average Number of Alcoholic Beverages Consumed Per Session of Autistic and Non-Autistic Adults

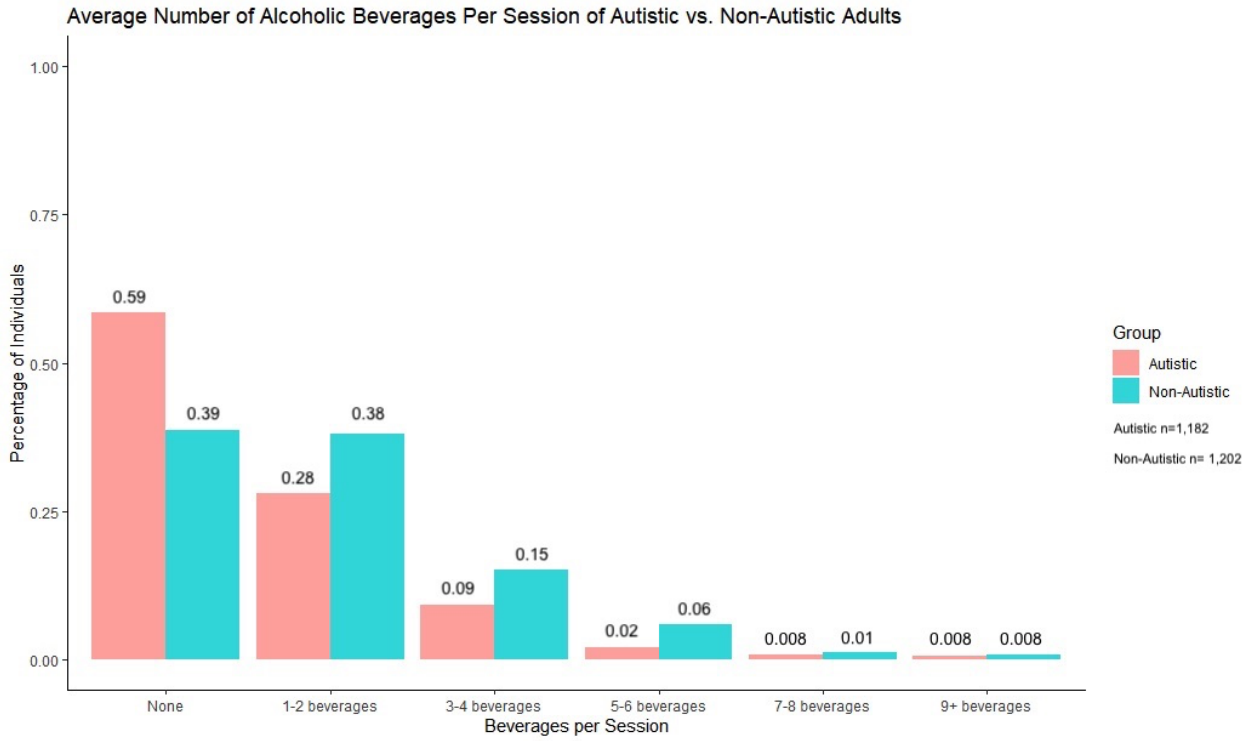

Figure S11: Average Smoking Frequency when Smoking Most of Autistic and Non-Autistic Adults

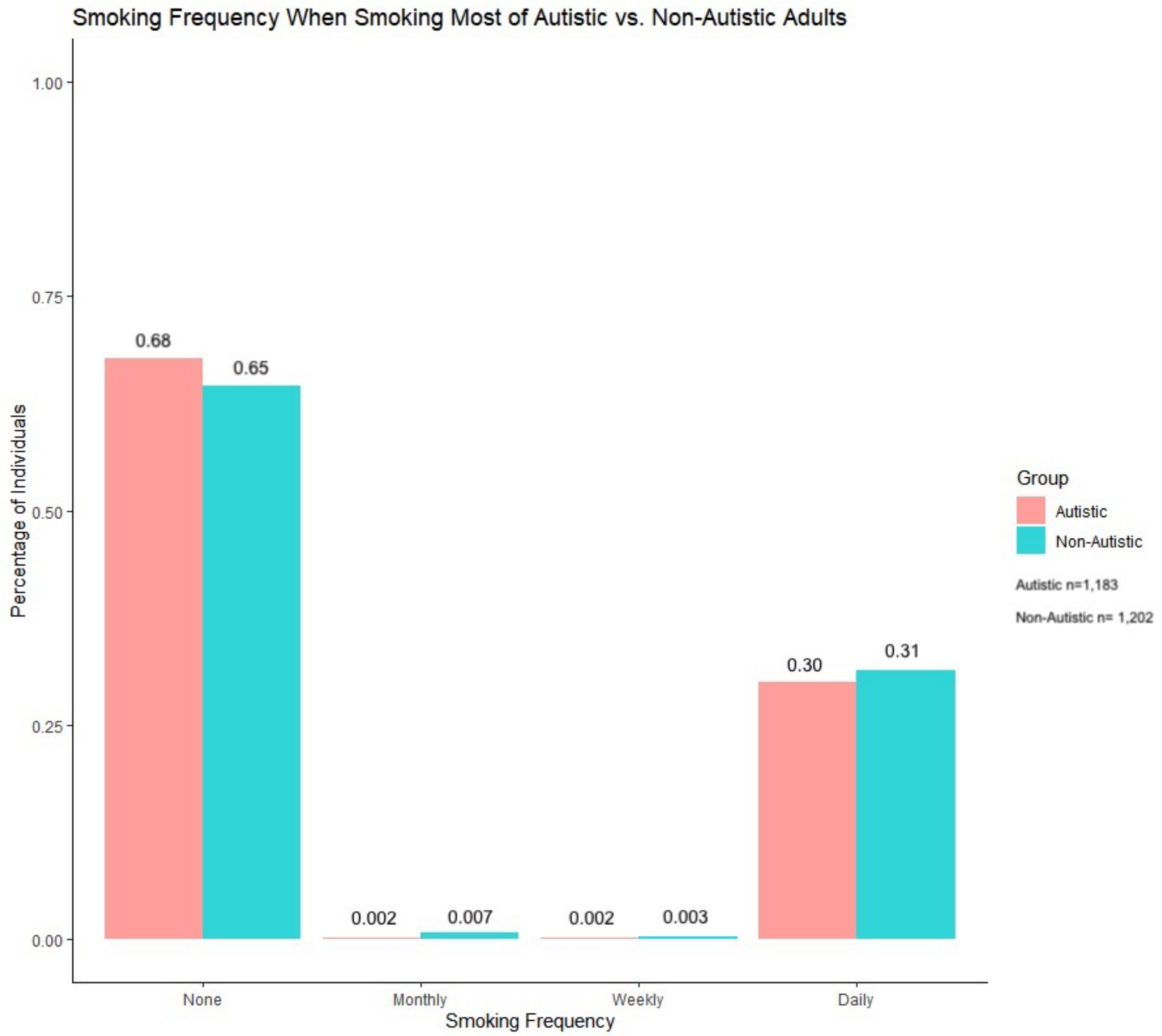

Autism and Physical Health Survey Protocol

I. Project Summary

The purpose of this study is to determine the typical physical health profile of adults with Autism Spectrum Conditions (ASC) and their first degree relatives. Particularly, this study will investigate if common medical conditions are more prevalent in individuals with ASC or their relatives. As this study attempts to be comprehensive in regards to the physical health profile, participants will be asked to complete questions on demographic information, physical health (including exercise, diet, sleep, immune function, disability, and social/ sexual history), and common medical conditions (including cancer; cardiovascular; respiratory; gastrointestinal; hormonal/reproductive; musculoskeletal; neurological; eye; ear, nose, and throat; liver and kidney; blood and lymph; skin; diabetes; and autoimmune conditions). Thus, our study aims to recruit individuals from all nationalities over the age of 16, with or without an Autism Spectrum diagnosis. They will complete an online, anonymous self-response survey will ask about the physical health and medical history of participants, as well as the family medical history of their first degree family members (parents, children, and siblings).

II. General Information

Title: Physical Health of Adults with Autism Spectrum Conditions and Their Relatives

Study Period: January 2018 - March 2019

Research Team: Professor Simon Baron-Cohen, Professor of Developmental Psychopathology, University of Cambridge, PhD; Miss Elizabeth Weir, MPhil Student, Autism Research Centre,

BA; Dr. Carrie Allison, Research Manager, Autism Research Centre, PhD; Ms. Paula Smith, Database Manager, Autism Research Centre

III. Rationale and Background Information

A great deal of research into Autism Spectrum Conditions (ASC) has focused on children. As such, there is very limited research on the physical health experience of adults with ASC. Beyond that, there are very few studies with a large enough sample size (at least 200 individuals with ASC) to accurately identify medical conditions that frequently co-occur with ASC—and far fewer that consider the health status of adults.

To date, there are three, large-scale studies that have attempted to identify common comorbidities of adults with ASC, specifically. Using retrospective data analysis of medical records from four hospitals in the United States, Kohane et. al (2012) found that individuals with ASC under the age of 35 are at greater risk for many comorbid conditions, including neurological, gastrointestinal, muscular, and metabolic conditions. Croen et. al (2015) did consider the health status of adults in Northern California (though less than 150 of the autistic participants were over the age of 50) and found higher prevalence of sleep, metabolic, and thyroid conditions— particularly among women. Most recently, using retrospective analysis of records in Quebec for individuals 24 and younger, Diallo et. al (2017) found increased risk of congenital abnormalities of the nervous system and heightened psychiatric comorbidity for individuals with ASC.

There are a few other studies on comorbidities of adults with ASC, though they have some methodological issues. Both Tyler et. al (2011) and Jones et. al (2016) found that autistic

individuals experienced higher prevalence of comorbidities and health care usage; however, these studies likely do not give very reliable information, as they had very small cohorts of autistic individuals with 108 and 92 participants, respectively. Additionally, Hsieh et. al (2014) conducted an analysis on obesity and weight-related factors of adults with intellectual disability that included a sub-group of autistic individuals; yet, the small sample size of 158 participants in the ASC subset likely prevented the experimenters from yielding statistically significant results.

These studies offer some evidence that individuals with ASC may experience a greater number of physical health issues; nevertheless, it is clear that future research should focus on the physical health experience of older adults with ASC. Thus, the proposed research expands beyond the existing literature in a number of ways. It aims to actively recruit adults (particularly those above 30 years of age) from all nationalities. Additionally, as very little is known about the health status of relatives of individuals with ASC, this study will be the first comprehensive physical health survey on ASC that asks participants to give information about the medical history of their first degree relatives (parents, full siblings, and children).

References

Croen, L. A., Zerbo, O., Qian, Y., Massolo, M. L., Rich, S., Sidney, S., Kripke, C., (2015). The health status of adults on the autism spectrum. *Autism*, *19*(7), 814–823. https://doi.org/10.1177/1362361315577517

Diallo, F. B., Fombonne, É., Kisely, S., Rochette, L., Vasiliadis, H.-M., Vanasse, A., ...Lesage, A. (2017). Prevalence and Correlates of Autism Spectrum Disorders in Quebec. *The Canadian Journal of Psychiatry*, 706743717737031. https://doi.org/10.1177/0706743717737031

Hsieh, K., Rimmer, J. H., & Heller, T. (2014). Obesity and associated factors in adults with intellectual disability. *Journal of Intellectual Disability Research*, *58*(9), 851–863. https://doi.org/10.1111/jir.12100

Jones, K. B., Cottle, K., Bakian, A., Farley, A., Bilder, D., Coon, H., McMahon, W. M. (2016). A description of medical conditions in adults with autism spectrum disorder: A follow-up of the 1980s Utah/UCLA Autism Epidemiologic Study. *Autism*, *20*(5), 551–561. https://doi.org/10.1177/1362361315594798

Kohane, I. S., McMurry, A., Weber, G., MacFadden, D., Rappaport, L., Kunkel, L., ... Churchill, S. (2012). The Co-Morbidity Burden of Children and Young Adults with Autism Spectrum Disorders. *PLOS ONE*, *7*(4), e33224. https://doi.org/10.1371/journal.pone.0033224

Tyler, C. V., Schramm, S. C., Karafa, M., Tang, A. S., & Jain, A. K. (2011). Chronic Disease Risks in Young Adults With Autism Spectrum Disorder: Forewarned Is Forearmed. *American Journal on Intellectual and Developmental Disabilities*, *116*(5), 371–380. https://doi.org/10.1352/1944-7558-116.5.371

IV. Study Goals and Objectives

This project aims to give a profile on the physical health of individuals with a diagnosis of ASC. Primarily, this includes determining if particular conditions are more prevalent in individuals with ASC, as compared to the general population. As nearly all of the previous research has been conducted on individuals younger than 35, this survey aims to recruit an older population (particularly by using targeted advertisements via our website, twitter, Cambridge Autism Research Database, as well as autism charities and support groups).

Additionally, it does not appear that any studies in this area have considered an international population; thus, our research aims to expand beyond this to include an international cohort. Furthermore, the vast majority of studies utilize retrospective analysis of medical records and, therefore, cannot ask about the medical conditions of family members. By utilizing a self-response survey, we are able to ask questions about first degree relatives of the participants, including parents, siblings, and children.

V. Study Design and Methodology

We will contact participants through a variety of avenues, including a press release from the Autism Research Trust, advertise on the Autism Research Centre's website contact via the Autism Research Centre's database, and targeted tweets sent out via Professor Baron-Cohen's personal twitter account. We will also use Facebook to advertise our study to members of the general population.

Individuals over the age of 16, with or without an Autism Spectrum diagnosis, will be asked to complete a detailed online questionnaire, prepared via Qualtrics. Before completing the survey, participants will complete our Participant Consent Form which will include a link to the Participant Information Sheet. After consenting to the anonymous survey, participants will be asked to complete questions on demographic information, physical health (including exercise, diet, sleep, immune function, disability, substance use, and sexual history), and common, non-communicable diseases and medical conditions. In addition, we will ask questions about the family medical history of the participants' first degree relatives.

Participants may be asked for follow-up questions, depending on their selections. For example, if a patient states that they have a lung condition, they will receive a follow-up question asking them to identify which of the following common lung conditions they have, such as asthma, chronic obstructive pulmonary disease, etc... However, participants will not see the larger list of conditions unless they select the ‘lung condition’ choice in the original, more general question. Similarly, the survey only asks about common cancers and will provide different options depending on the participant’s selection for biological sex (ie testicular, ovarian, etc...).

As such, though the survey appears lengthy, each survey participant should be prepared to answer approximately 50-60 questions. We expect that the survey will take participants 10-20 minutes to complete. It is important to note that the survey is anonymous and will not contain any patient identifiable information. As such, we will not contact participants who have completed the survey to ask additional questions. Participants will be presented with a link to a separate contact form, if they would like to hear the results of the survey.

VI. Consent

Before completing any other survey questions, participants must confirm that they are at least 16 years of age and that they read and understand the expectations of the study, as outlined in the Participant Information Sheet. If an individual selects the option that they do not consent to completing the following questions, they will not be asked for any other information and the survey will end. As we are not collecting personally identifiable data, it will not be possible for us to identify individual responses to the survey. As such, it may not be possible for us to exclude an individual’s partially or fully completed questionnaire if they contact us afterwards

and wish to be excluded. This information is clearly explained to participants via the information sheet and consent form.

VII. Population

We hope to recruit as many participants as possible, though we would like to have at least 1,000 participants (500 cases, 500 controls). The existing literature is very limited on adults aged 50 or older; the largest study that reports age distribution indicated that less than 150 subjects were 50 years or older. We hope to recruit a greater number of older adults than this, but also do not want to be unrealistic about the number of participants that can be expected for a survey of this type. This expectation seems reasonable, as another recent survey on Autism and Vulnerability conducted at the Autism Research Centre yielded a similar distribution of responses.

As the advertisement methods for this study may bias the control group towards those that score higher for autistic traits than the general population, we have included a copy of the AQ-10 as a safeguard. It will only be filled out by individuals without a formal autism spectrum diagnosis and is presented to participants as an assessment of personality.

In addition to asking demographic questions (age and sex), we will be asking individuals if they have a diagnosis of a syndromic form of autism (fragile X syndrome, tuberous sclerosis, etc...) in order to determine if this group skews the sample and include or exclude them from our results, accordingly. There are specific questions in the survey, designed to determine if any of the above criteria applies; all participants must complete these questions in order to complete the survey.

VIII. Statistical Analyses

We will be performing a case-controlled study and will survey both autistic and non-autistic individuals. In addition, as indicated above, we will exclude participants based on diagnosis of a syndromic form of autism in analyses related to physical health outcomes. We will either exclude those without a diagnosis of ASC but a high AQ score or we will make them into a third, intermediate group for analysis.

The strategies that we employ regarding statistical analyses will depend on our sample size. However, we will likely use Fisher's exact test to establish if those with ASC are more likely to develop particular medical conditions. Depending on the results of these initial analyses, we may use logistic regression to control for confounding and determine if there are any additive effects regarding risk factor (e.g. do high BMI, prediabetes/ diabetes affect risk of particular cancers).

IX. Follow up

Participants will be told that the survey asks about physical health overall— though the primary purpose of the study is to collect data on the patient and family medical history for comorbid medical conditions. The survey asks about overall health (including diet, exercise, sleep, immune function, disability, and social/ sexual history) in order to avoid response bias in which the cohort is overly representative of individuals with comorbidities.

Additionally, as we are not recording personally identifiable information as a part of the survey, we will not be able to contact participants in the study regarding the results of the survey—unless they provide their email address in the form attached to the Information Sheet. Please note that

we will not be able to link the submissions to the form to the survey responses. As such, the survey responses will not contain any personally identifiable information.

X. Ethical Issues

It is possible that patients may be distressed or made uncomfortable by the more sensitive questions about social and sexual history. In order to mitigate this risk, there is a disclaimer before the beginning of this section indicating that all questions are entirely optional to complete and that they can feel free to skip any questions for which they do not wish to provide answers. Additionally, we remind participants that their responses are entirely anonymous and we have no means of linking their responses back to them. The information sheet also reminds patients that their participation is entirely optional and that they can suspend their participation at any point by exiting the survey. If any individual reaches out to us to indicate that they became distressed or uncomfortable as a result of the questionnaire, we will determine the nature of the concern and resolve it appropriately.

It is also possible that patients may be distressed or made uncomfortable by recounting their personal and family medical history, as the survey asks about a number of serious medical conditions. We have attempted to put participants at ease by clearly stating this, along with our expectations for participants, on the Information sheet and consent question at the beginning of the survey. We make it clear in the information sheet that participation in the study is entirely voluntary. If any individual reaches out to us to indicate that they became distressed or uncomfortable as a result of the questionnaire, we will determine the nature of the concern and resolve it appropriately.

Another possible ethical issue is that participants are providing information about their relatives—who have not consented to participate in the study. However, as we are not collecting any personally identifiable data from either the participant or the relative as a part of this survey, we do not feel that this outweighs the benefits of conducting the survey.

The final, possible ethical issue is that we may not be able to retroactively exclude partial or full survey responses from analysis if a participant wishes to be excluded after beginning the survey. This is simply because we are not collecting any personally identifiable data as a part of the questionnaire. As such, we will not be able to easily link an individual back to their survey responses. To make this evident to participants, we have indicated this on the Information sheet, consent form, and at the beginning of the survey.
